# Toward an Integrative Understanding of *Dianthus gratianopolitanus*: Genetics, Physiology, Ecology, and Conservation

**DOI:** 10.3390/ijms27219504

**Published:** 2026-10-24

**Authors:** Katarzyna M. Zielińska, Izabela Kołodziejczyk, Ewa Michalska

**Affiliations:** Department of Biogeography, Paleoecology and Nature Conservation, Faculty of Biology and Environmental Protection, University of Lodz, Banacha 1/3, 90-237 Lodz, Poland; katarzyna.zielinska@biol.uni.lodz.pl (K.M.Z.); ewa.michalska@edu.uni.lodz.pl (E.M.)

**Keywords:** Cheddar pink, population genetics, endangered species, chloroplast genome

## Abstract

Effective conservation of endangered species under increasing anthropogenic pressure requires a thorough understanding of their biology. However, the growing specialization of modern biology has fragmented botanical science into separate disciplines, characterized by its own terminology, methods, and conceptual frameworks. As a result, researchers working on the same taxon may struggle to integrate findings. Yet, some of the most important biological questions arise precisely at the intersection of studies in genetics and physiology and field ecology. This review synthesizes current knowledge on the genetics, physiology, and ecology of *Dianthus gratianopolitanus* (Cheddar Pink). Despite its widespread horticultural use, the species remains rare in its natural habitats and is red-listed in multiple countries. The aim of this review is to identify integrative research directions relevant to its effective conservation. The published literature on *D. gratianopolitanus* was reviewed, focusing on chloroplast genomics, population genetics, physiological adaptations, population ecology, and habitat requirements. Attention was given to research questions emerging at the intersection of disciplines. *D. gratianopolitanus* displays apparent ecological flexibility alongside strong microhabitat dependence; population-level differentiation reflects a combination of edaphic adaptation, phenotypic plasticity, and biogeographic history, while chloroplast genomic analyses reveal positive selection in photosynthesis- and stress-related genes, whose functional significance remains to be experimentally validated. Major knowledge gaps persist in the mechanisms translating environmental conditions into physiological performance, the relative contributions of genetic fixation versus plasticity to local adaptation, long-term demographic dynamics, and the criteria for genotype-informed conservation translocations. This review proposes that bridging ecological, physiological, genomic, demographic, and biotechnological research is both feasible and necessary for the long-term conservation of the species.

## 1. Introduction

Each plant species raises countless research questions. However, increasingly specialized analyses fragment botanical science into isolated domains with discipline-specific languages. Even scientists studying the same taxon may struggle to integrate findings across disciplines. Field ecologists, who describe distribution and habitat, often find it challenging to integrate results from laboratory-focused research in genetics, proteomics and physiology. Conversely, mathematical models analyzing species’ niches and distributions based on field occurrence data pose challenges for laboratory-based physiologists. The most compelling questions, however, emerge at the intersection of these fields. Interdisciplinary research integrating diverse scientific approaches is essential for understanding species biology realistically and for implementing effective conservation strategies, particularly for endangered taxa [1].

This article examines *Dianthus gratianopolitanus* Vill. (Cheddar pink), a species protected or red-listed across multiple European countries (Poland, Germany, Czech Republic, Switzerland, France, United Kingdom) and characteristic of xerothermic or rocky grassland communities, many of which are protected within the Natura 2000 network [2,3,4,5]. Despite being easily propagated and widely available in horticulture, this species remains rare in nature. While *D. gratianopolitanus* has attracted substantial scientific interest, few studies adopt interdisciplinary frameworks [6,7,8].

This review synthesizes current knowledge on *D. gratianopolitanus*, with primary emphasis on molecular biology, population genetics, and physiology, framed within the ecological context of the species’ environmental requirements and distribution. Aspects of morphological structure are not addressed. Its aim is to pose integrative research questions linking molecular and physiological mechanisms to ecological patterns—questions that arise from comparing what is known about the species’ external environmental conditions with what is known about how its biological machinery operates at the molecular and physiological levels. The article is written to be accessible to scientists from various fields.

## 2. Literature Search and Study Selection

To achieve the aim of this review, the literature concerning *Dianthus gratianopolitanus* was systematically searched and screened according to predefined inclusion and exclusion criteria (Table 1). During the initial screening, the publication year, title, and abstract were assessed to identify potentially relevant studies. The full texts of potentially eligible publications were subsequently assessed against the predefined eligibility criteria.

The systematic literature search was conducted in accordance with the Preferred Reporting Items for Systematic Reviews and Meta-Analyses (PRISMA) 2020 guidelines. Four primary scientific databases were searched: PubMed, Scopus, Web of Science, and Embase. The search was complemented by Google Scholar and ScienceDirect to identify additional relevant publications and references not retrieved through the primary databases. The literature search was conducted up to and including 7 October 2026.

The search strategy combined controlled vocabulary, where applicable, with free-text keywords related to *Dianthus gratianopolitanus*. The search included the accepted scientific name *Dianthus gratianopolitanus* Vill., its taxonomic synonym *Dianthus caesius*, and common names used in the literature, including “Cheddar pink”, “Pfingst-Nelke”, and “Pfingstnelke”. These terms were combined with keywords referring to the main aspects of the biology, ecology, distribution, conservation, reproduction, genetics, taxonomy, phylogeny, physiology, adaptation, and molecular biology of the species.

An example of the search strategy used in PubMed was: (“Dianthus gratianopolitanus” OR “Dianthus caesius” OR “Cheddar pink” OR “Pfingst-Nelke” OR “Pfingstnelke”) AND (taxonomy OR phylogeny OR genetics OR population OR ecology OR distribution OR conservation OR reproduction OR pollination OR germination OR seed OR “climate change” OR habitat OR morphology OR physiology OR adaptation OR chloroplast OR genome OR “molecular markers” OR “genetic diversity” OR reintroduction OR restoration). Equivalent search strategies were adapted to the search syntax and indexing requirements of the individual databases.

Because relevant information on the molecular biology and genetic characteristics of *D. gratianopolitanus* is also available through nucleotide sequence repositories, the National Center for Biotechnology Information (NCBI) database was additionally consulted. The Basic Local Alignment Search Tool (BLAST, version 2.17.0) was used as a complementary resource for the identification and comparison of nucleotide sequences associated with *D. gratianopolitanus*. NCBI and BLAST were considered supplementary sources of molecular information rather than bibliographic databases.

In addition to database searches, the reference lists of relevant publications were manually screened to identify additional studies potentially meeting the eligibility criteria. Particular attention was given to publications addressing the taxonomy, distribution, genetic diversity, reproductive biology, ecological requirements, habitat associations, physiological adaptations, conservation, reintroduction, and molecular characteristics of *D. gratianopolitanus*. Relevant monographs, dissertations, red lists, species conservation documents, and other authoritative sources were also considered where they provided original or otherwise relevant information concerning the species.

Publications were considered eligible when they (i) focused directly on *D. gratianopolitanus* or provided species-specific data for this taxon; (ii) addressed at least one aspect of its taxonomy, biology, ecology, distribution, population biology, reproduction, genetics, physiology, adaptation, conservation, or molecular characteristics; and (iii) provided sufficient information to evaluate the reported findings. Publications dealing with the genus *Dianthus* as a whole were included only when species-specific information concerning *D. gratianopolitanus* could be clearly identified. Studies concerning other *Dianthus* species without relevant information on *D. gratianopolitanus* were excluded.

The following types of sources were excluded: (i) publications unrelated to *D. gratianopolitanus*; (ii) studies in which species-specific information could not be separated from data concerning other taxa; (iii) duplicate publications or duplicate records of the same study; (iv) publications for which the full text or sufficient bibliographic information was unavailable; and (v) sources that did not provide information relevant to the objectives of the review. Where several publications reported substantially overlapping information, the most complete or primary source was preferentially considered.

## 3. Basic Information About the *Dianthus gratianopolitanus* Species

**Figure 1:**
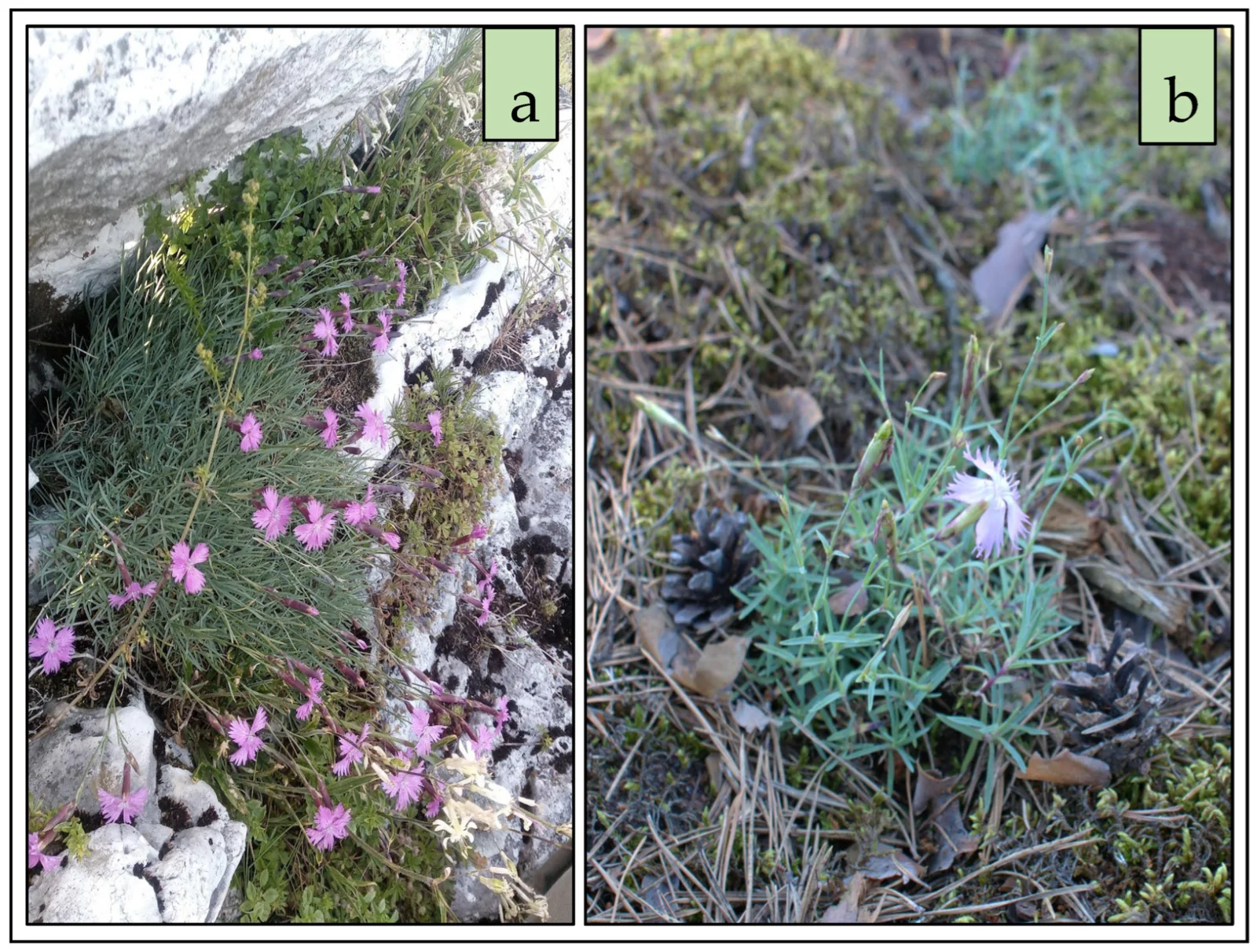
(**a**) *Dianthus gratianopolitanus* on limestone rocks—natural site (photo: K.M. Zielińska, Góra Zborów Reserve, Poland, 3 June 2022), and (**b**) in a pine forest—an individual planted as part of strengthening the natural population (photo: K.M. Zielińska, near the village Kurnos Drugi, Poland, 8 June 2016).

*Dianthus gratianopolitanus* Vill. (*Caryophyllaceae*) (Figure 1) is a long-lived perennial that forms cushions or mats, particularly in grasslands developed on rocky ridges and limestone outcrops, but also on soils derived from serpentine and siliceous bedrock, for example, inland dunes covered with pine forest [8,9,10,11].

In addition to its cushion-like growth form, the species possesses several xerophytic characteristics, for example, tubular cuticular wax and relatively small non-stomatal water loss [12]. The overwintering leaf rosettes are exposed not only to summer drought and heat but also to winter desiccation caused by frost [2]. It can be considered a stress-tolerant species in the sense of Grime’s CSR framework [13], a species that thrives under extreme conditions with limited resources [2].

The plant reproduces via insect-pollinated flowers borne on 10–25 cm tall shoots, but also spreads through the vegetative growth of creeping shoots [10,11]. It is long-lived, yet has low production and reproduction rates. The flowering period of *D. gratianopolitanus* typically extends from late April to late June [14]. However, there are clear indications that individual clumps—and often all or most clumps on a given site—do not reach full flowering every year [2]. In no case has limited reproductive success been attributed to a shortage of suitable pollinators [15]. Cheddar pink produces capsules that contain numerous seeds; the potential for long-distance dispersal is, however, severely limited because the seeds fall quite close to the mother plants and rarely find microhabitats suitable for germination [2,8].

*Dianthus gratianopolitanus* is a central European endemic with a highly fragmented distribution. Its distribution center lies along the Jura Mountains (French, Swiss, and Swabian Jura), with more isolated populations in the low mountain ranges of central and eastern France; southern Belgium; Germany; Bohemia; and on lowland sites—particularly in Poland. The most isolated population is found in England [16,17]. Schöne (2025 [8]), in a doctoral dissertation, conducted phylogeographic research on the spatial structure of genetic variation. Her conclusions support a scenario of more or less continuous postglacial distribution, followed by increasing fragmentation that produced the present-day pattern of largely isolated populations [8]. Nevertheless, highly isolated populations are neither smaller nor reproductively weaker, and levels of genetic variation remain stable within them [18]. This suggests that the species is naturally adapted to functioning under conditions of severe habitat fragmentation. Due to its capacity for vegetative reproduction and its limited potential for dispersal via diaspores, *D. gratianopolitanus* is evidently dependent primarily on long-term survival at relict and largely isolated sites [2].

## 4. Ecology

### 4.1. Bedrock and Soils

*Dianthus gratianopolitanus* occurs mainly in dry rocky habitats with shallow soils over limestone. However, it also grows on non-calcareous rocks, including basalt, phonolite, phyllite, granite, syenite, conglomerate, sandstone, diabase, porphyry, and gneiss [16]. Calcareous bedrock predominates in the southern part of the species’ range, while siliceous bedrock is more common in the northern part. Schöne (2025 [8]) demonstrated that populations from calcareous and siliceous substrates differ in growth and reproductive strategies, but found no evidence of strict soil specialization. Overall, the species exhibits considerable ecological plasticity, enabling it to colonize a variety of substrate types.

The species can persist on the outermost edges of rock outcrops that are extremely poor in humus and in podzolic soils typical for Pine forests [2,11]. In germination experiments, seeds of *D. gratianopolitanus* germinated across a pH range of 5 to 9, with maximum germination at pH 7. The authors concluded that in moist soils, pH may not be a limiting factor for germination [19]. The ability to germinate effectively beyond the optimal pH may enhance the species’ potential to colonize habitats with diverse soil chemistry.

### 4.2. Light

Numerous field observations regarding light requirements were provided [2]. The species is considered photophilous (light-demanding): dense cushions develop in full sun, while vitality declines in shade. Plants growing beneath shrubs on rocks or under a tree canopy show altered morphology and flower sparsely. It was also noted that on north-facing rocks, the species occurs only in exposed positions, while on south-facing rocks, it tolerates, or even prefers, light shading from woody plants—clearly indicating that light is a limiting factor. Furthermore, the occurrence of the species in exposed positions on south-facing slopes can be attributed to higher altitude [20]. Thus, the limiting factor on exposed south-facing slopes may not be excessive irradiance itself, but high temperatures. This interpretation remains hypothetical and requires direct experimental testing.

Light also appears to be important for seed germination, particularly at lower temperatures. Kołodziejek et al. (2018) experimentally demonstrated that at 15 °C/5 °C (day/night), germination is severely limited in darkness, whereas at 25 °C/15 °C it reaches 88–90%. Germination was consistently higher under light than in darkness across all thermal regimes tested [19].

### 4.3. Temperature and Precipitation

Koch et al. (2021 [7]) found a relationship between the onset of flowering in *D. gratianopolitanus* and the mean annual temperature, as well as the accumulated daily temperature sum from February to June. They observed a shift in the flowering peak of up to three weeks depending on the early-season temperature sum. Unfortunately, earlier flowering was accompanied by a reduction in flower number and seed set, which the authors attribute to the negative effects of higher spring temperatures—both on the total number of flowering individuals and on risk to pollinator synchrony [7].

Winter temperatures may also influence flowering intensity. In experimental work on the garden cultivar *D. gratianopolitanus* ‘Bath’s Pink’, Padhye & Cameron (2008) confirmed a near-obligate vernalization requirement: the shortest cold treatment that stimulated 100% of plants to flower was three weeks at 5 °C, although plants vernalized for longer periods produced more flowers [21]. It should be noted, however, that ‘Bath’s Pink’ is a horticultural selection. Whether wild populations share the same threshold requires direct experimental verification. Reduced flowering necessarily entails reduced seed production, a consequence also documented by Koch et al. (2021), who showed that elevated temperatures reduce not only flower number but also seed set [7].

Temperature also proved to be a key determinant of germination success. Kołodziejek et al. (2018 [19]) recorded germination rates of 97–99% at 25 °C/15 °C (day/night, under light), dropping to 56–60% at 20 °C/10 °C and to 34–38% at 15 °C/5 °C. Warmer conditions not only increased the proportion of germinating seeds but also accelerated the germination process. Cold stratification (four months at 4.5–5.1 °C) did not significantly affect the final germination percentage, but did increase the germination rate (i.e., the speed of germination) [19].

The seeds of *D. gratianopolitanus* are non-dormant—that is, they germinate immediately when suitable conditions are encountered—and orthodox, meaning they tolerate drying and long-term storage well [19,22]. Germination is primarily governed by water availability. Notably, *D. gratianopolitanus* seeds can germinate under comparatively low soil moisture conditions relative to other species [19,23], which is consistent with the species’ typical occurrence in dry, exposed habitats. However, for young plants established through active conservation means (transplanting), adequate water supply during the establishment phase is critical [22]. This is also described by Banzhaf et al. (2009) in their observations of naturally occurring seedlings—summer drought can be a serious threat to young plants [2].

As with many plant species, studies of *D. gratianopolitanus* have often examined individual environmental factors in isolation, providing detailed descriptions of single-factor requirements. Analyses accounting for the synergistic effects of multiple factors are largely limited to mathematical modeling—specifically, species distribution modeling (SDM). SDM attempts to characterize the climatic conditions at locations where the species actually occurs, set against the background of overall climate variability within the geographic range. This approach captures synergistic effects among climatic variables but is prone to error because it cannot account for micro-scale variability, for example, the temperature differences between rock outcrops and surrounding forest are not captured by gridded climate data. A key strength of the method is that variables used in SDM analyses describe temperature and precipitation not only as averages, minima, and maxima, but also through biologically meaningful combinations—for example, precipitation of the driest month. A standard set of 19 such bioclimatic variables (BIO1-BIO19) is commonly used (e.g., [24]). For *D. gratianopolitanus*, the variables most important for explaining the geographic distribution were analyzed in the doctoral dissertation of Schöne (2025 [8]). Precipitation of the warmest quarter (BIO18), temperature seasonality (BIO4), precipitation of the driest month (BIO14), and minimum temperature of the coldest month (BIO6) proved to be the most important [8]. When analyses were conducted separately for populations on calcareous and siliceous (volcanic + sandy) bedrock types, some differences emerged. It must be remembered that populations on different substrates occupy climatically distinct portions of the species’ range, reflecting the species’ considerable climatic plasticity rather than a strict edaphic-climatic coupling.

### 4.4. Biotic Relations

In the German literature, *D. gratianopolitanus* is the characteristic species of the association *Diantho gratianopolitani-Festucetum pallentis* Gauckler 1938 [25] of the class *Sedo-Scleranthetea*. The same community is distinguished in Switzerland and France [26,27]. In the Polish literature, it is characteristic of the alliance *Seslerio-Festucion duriusculae* Klika (1931) 1948 of the class *Festuco-Brometea* [28]. Across different countries, grasslands on rock outcrops may be classified under different syntaxonomic systems, but in each case, *D. gratianopolitanus* is characteristic of thermophilous grassland communities with *Festuca pallens*. These are the communities associated with the species (*Dianthus* functions here as a characteristic species, diagnostically linked to these communities), but they are not the only ones in which it occurs. In the Polish lowlands, it is most often found in pine forests of the *Dicrano-Pinion* alliance [11]. In pine forests it was also noted in Germany [2].

*D. gratianopolitanus* is apparently a very weak competitor [2]. It appears to tolerate drought better than most of its herbaceous competitors, which may explain its persistence in the harshest microhabitats. At the edges of rocky outcrops and plateaus, humus is continually washed and blown away, preventing the development of thicker humus layers. Where the humus layer becomes too thick, slightly more demanding competitors gain the advantage. In areas shaded by trees, the accumulation of leaf litter and tall and dense moss clumps have been identified as factors negatively affecting *D. gratianopolitanus* [2]. However, the species appears to cope better with root competition from woody plants than with dense herbaceous or grass competitors.

*D. gratianopolitanus* contains saponin glycosides, which provide some protection against grazing by wildlife. Nevertheless, winter damage by small rodents was observed [2]. It is a typical butterfly-pollinated plant, attracting pollinators with both its showy petals and fragrance. Visitors include hawk moths, butterflies, and diurnal and nocturnal noctuid moths, giving the species a wide range of potential pollinators [15]. The relationship with the genus *Hadena* (*Noctuidae*) is very interesting, as it pollinates flowers, but also lays eggs in them, so that the caterpillars can feed on the fruits and even the leaves [22].

## 5. Genetics

### 5.1. Chloroplast Genome Organization, Genetic Variation, and Molecular Evolution

The year 2015 was a turning point in carnation analysis, as a plastome from the species *D. gratianopolitanus* was reported for the first time [29,30]. By 2023, only nine chloroplast genome (cpDNA) sequences of *D. gratianopolitanus* varieties were included in the National Center for Biotechnology Information Database (https://www.ncbi.nlm.nih.gov/ accessed on 2023). Meng et al. [31] and Lin et al. [32] addressed this gap and examined the cpDNA of four species: *D. caryophyllus*, *D. longicalyx*, *D. moravicus*, and including nine varieties of *D. gratianopolitanus*: dg1602 (LN877389), dg1869 (LN877391), dg3051 (LN877395), dg2134 (LN877392), dg1856 (LN877390), dg2769 (LN877393), dg1255 (LN877387), dg1370 (LN877388), and dg2868 (LN877394; [31]), and independently, *D. gratianopolitanus* (LN877392), *D. chinensis* (NC_053731), and *D. longicalyx* (NC_050834; [32]), described with the accession numbers and selected in the BLAST search (https://blast.ncbi.nlm.nih.gov/ accessed on 2023) according to the nucleotide sequence similarity to determine their phylogenetic relationships.

The analyses revealed that the cpDNA sizes range by a value of 149,665 bp in *D. gratianopolitanus* (LN877392, [32]), also from 149,474 to 149,735 bp (with slight variation), with GC content between 36.29 and 36.33%, relatively high in inverted repeats regions (IR regions), which were longer than in other species [31]. The research allowed identifying also 124–125 genes (which place *D. gratianopolitanus* among the other studied species), including 84 protein-coding genes (Table 2).

Microsatellite repeat sequence (SSR) analysis revealed 142, 150, 141, 149, 144, 142, 140, 146, and 146 SSRs in *D. gratianopolitanus* varieties (Table 2), respectively, which was close to *D. longicalyx* with 145 and *D. moravicus* with 147 SSRs, but not overlapping with 134 SSRs with 13 combined types in *D. caryophyllus* [31].

In the plant material, six types of SSRs (Table 3), mononucleotide and dinucleotide repeats, occurred most frequently, from 55 to 67 copies. In turn, trinucleotide and tetranucleotide repeats were analogous, and pentanucleotide repeats did not occur as frequently (the most frequently occurring in each of the studied variants averaged approximately 4). One copy each of the hexanucleotide repeats was found in cpDNA three variants of *D. gratianopolitanus*: dg1602, dg2769, and dg3051; also in *D. longicalyx*, but *D. caryophyllus* and other plant materials did not have any [31].

Multiple sequence alignment analysis of *D. gratianopolitanus* cultivars discovered 49, 49, 49, 49, 49, 49, 49, 42, 44, 42 repeat sequences, respectively (compared to *D. caryophyllus* with 42 repeat sequences, *D. longicalyx* with 49 and *D. moravicus* with 47). Five species contained very few complementary repeat sequences, with *D. gratianopolitanus* (dg2769) containing the most, although up to four. It should be noted, however, that the average number of forward reached 14, for reverse 12, and in palindromic repeats 20, with the length of palindromic repeats (forward) as 20–29 bp (over 59 bp determined in *D. gratianopolitanus* varieties dg1602 and dg3051) and followed as 30–39 bp [31].

The repeat sequences found in *D. gratianopolitanus* were consistent with those of the other analyzed material. The differences in the genomes were minimal, but research [31] revealed significant variations in conserved non-coding (CNS), rRNA and tRNA coding sequences.

Significant gene (Table 4) substitutions were observed in the boundary regions: IR, the large single-copy region (LSC), and the Small Single-Copy region (SSC) showed the greatest difference in *D. gratianopolitanus* cultivars in the 1062 bp value. The *psbA* gene [31] of photosystem II encodes the D1 protein, which plays a key role in electron transport during photosynthesis [33], at the LSC location in the dg1869 variant. The same was for the transfer RNAs gene, *trnH*, in the other cultivars [31]. The *trnH* gene encodes tRNA for histidine [34].

In *D. gratianopolitanus* cultivars, variant sites in the protein-coding sequences were located mainly in the genes: the *atpF* gene encodes the ATP synthase subunit I, which is involved in ATP production during photosynthetic phosphorylation—in the dg1255 and dg1370 cultivars [31,35]; the *clpP* gene encodes the proteolytic subunit of the ATP-dependent Clp protease complex, which is involved in protein turnover and quality control in chloroplasts—in dg1602 and dg2868 [31,36]; the *petD* gene encodes subunit IV of the cytochrome b_6_f complex, which plays an essential role in electron transport and proton translocation during photosynthesis [37]; and the *ycf3* gene encodes a photosystem I (PSI) assembly factor that is essential for the biogenesis and functional stability of the PSI complex in chloroplasts [38]—in dg3051 [31].

Considering the differences, the largest were revealed in the *atpF*, *clpP*, and *ycf3* genes, with a significantly aberrant *rpl16* in *D. caryophyllus* [31]. The *rpl16* gene encodes the ribosomal protein L16, a component of the chloroplast 50S ribosomal subunit, which is essential for protein synthesis. In many plants, *rpl16* contains a group II intron that requires nuclear-encoded splicing factors for proper RNA maturation [39,40].

The species also did not have deletions in these genes, a feature revealed in the other material studied. In turn, the *rpl2* gene in *D. gratianopolitanus* had the same features as in the other analyzed material. Another gene, *rps19*, in *D. gratianopolitanus*, within varieties, had the same length and location as in the other species, except for *D. caryophyllus*, in which LSC/IRa contained the *ycf2* gene [31]. The *rps19* gene encodes the ribosomal protein S19 of the chloroplast small ribosomal subunit, playing a key role in 16S rRNA binding and translational initiation, and is frequently located at the LSC/IR border in plant plastomes [41].

In contrast, the *ycf1* (hypothetical chloroplast reading frame 1) gene encodes one of the largest and most functionally important proteins in the chloroplast genome. Although initially classified as an open reading frame of uncertain function, subsequent research demonstrated that *ycf1* encodes a component of the TIC (Translocon at the Inner Chloroplast membrane) complex, which mediates protein import from the cytosol into the chloroplast stroma.

Specifically, *ycf1*, which is essential for the translocation of nucleus-encoded preproteins and is required for chloroplast biogenesis and viability in most land plants [42], in the IRb region was the same length in the studied varieties and species, but with a 3-bp difference in *D. caryophyllus*. However, compared to *D. gratianopolitanus* dg1869, *D. caryophyllus* possessed highly similar gene sequences [31].

Meng et al. (2023 [31]) also examined the Relative Synonymous Codon Usage (RSCU). They found that the RSCU value of synonymous codons varied significantly. The vast majority of amino acids (except tryptophan and methionine) contained synonymous codons (leucine and arginine having the highest codon usage with the same RSCU values). When comparing plant variants, they found that more than 50% of codons had an RSCU above 1.0, with a preference for codons ending in A or U [31].

It should be mentioned that an RSCU value of 1.0 indicates that the codon is used at the same frequency as its synonymous counterparts. Values above 1.0 suggest that the codon is overrepresented, whereas values below 1.0 indicate underrepresentation [43]. In addition, the K_a_/K_s_ ratio (also referred to as dN/dS) was examined [31]. It is a comparative genomics metric used to assess the selective pressure acting on protein-coding genes. It represents the ratio between the number of nonsynonymous substitutions (those altering the amino acid sequence; K_a_) per nonsynonymous site and the number of synonymous substitutions (those not altering the amino acid sequence; K_s_) per synonymous site. A ratio greater than 1 indicates positive (adaptive) selection, a ratio less than 1 suggests purifying (negative) selection, while a ratio close to 1 reflects neutral evolution [44]. The analysis [31] revealed that the K_a_/K_s_ ratio in *D. gratianopolitanus* cultivars was similar. In a large portion of the protein-coding gene pool, it was below 1.0, indicating that these genes were subject to purifying selection.

In all these plant variants, the *atpB*, *ndhF*, *psbB*, and *ycf1* genes had ratios above 1.0, which in this case indicates the occurrence of positive selection. The *atpB* gene encodes the beta subunit of ATP synthase (CF_1_-β), a key enzyme complex located in the thylakoid membrane of chloroplasts. This subunit plays an essential role in ATP synthesis during photophosphorylation, where it participates in the catalytic conversion of ADP and inorganic phosphate into ATP, driven by the proton motive force generated by photosynthetic electron transport [45,46]. In *D. gratianopolitanus*, a K_a_/K_s_ ratio was greater than 1.0, suggesting that it is subject to positive selection and may play a role in the adaptive optimization of photosynthetic ATP production [31,45,46]. In *D. gratianopolitanus*, the *ndhF* gene encodes the subunit F of the NADH dehydrogenase-like (NDH) complex, located in the thylakoid membrane of the chloroplast. This complex is functionally analogous to mitochondrial complex I, playing a critical role in cyclic electron transport around PSI and chlororespiration. The NDH complex contributes to maintaining redox balance and protecting the photosynthetic apparatus under environmental stress conditions such as drought, high light, or temperature fluctuations [47,48].

It was shown that a K_a_/K_s_ ratio exceeds 1.0, suggesting that this gene is under positive selection and may contribute to adaptive adjustments of photosynthetic efficiency, light capture and energy transfer [31,47,48,49,50]]. The *psbB* gene encodes the CP47 protein, a core antenna component of PSII, embedded in the thylakoid membrane of chloroplasts. This protein binds chlorophyll and carotenoids, functioning as a light-harvesting subunit that transfers excitation energy to the PSII reaction center. CP47 plays an essential role in the assembly and stabilization of PSII, ensuring efficient electron transport from water oxidation to plastoquinone reduction during photosynthesis [49,50].

The *ycf1* gene exhibited a K_a_/K_s_ ratio above 1.0, indicating that it is under positive selection and may contribute to adaptive evolution in chloroplast protein import and genome stability [31,51,52].

Of particular note is the *rpoC2* gene, with a ratio also above 1.0 only in *D. gratianopolitanus* dg1869 and dg2134. The *rpoC2* gene encodes the β′ (beta-prime) subunit of the plastid-encoded RNA polymerase (PEP), an enzyme complex responsible for transcription of photosynthetic and other chloroplast genes. The PEP complex is structurally related to the bacterial multisubunit RNA polymerase and consists of subunits encoded by the *rpoA*, *rpoB*, *rpoC1*, and *rpoC2* genes. The *rpoC2* subunit plays a crucial role in enzyme assembly and promoter recognition, directly influencing transcriptional regulation of chloroplast gene expression. The *rpoC2* gene showed a K_a_/K_s_ ratio above 1.0, suggesting that it is under positive selection and may contribute to the adaptive evolution of chloroplast gene expression [31,53,54]. The *rpoA* gene encodes the α (alpha) subunit of the plastid-encoded RNA polymerase (PEP), a multisubunit enzyme complex responsible for transcription of chloroplast genes, particularly those involved in photosynthesis and plastid gene expression. The *rpoA* product forms part of the PEP catalytic core, which also includes subunits encoded by *rpoB, rpoC1*, and *rpoC2*. Within this complex, the α subunit participates in enzyme assembly, promoter recognition, and interaction with transcriptional regulators, making it a key component in the regulation of plastid transcription.

Similarly to *rpoC2*, the level of the *rpoA* K_a_/K_s_ ratio was very high in *D. gratianopolitanus* dg1602, dg3051, and dg2769, indicating strong positive selection, suggesting that it is under positive selection and may play an adaptive role in the regulation of chloroplast gene expression [31,55,56]. An important aspect of the obtained results was that none of the protein-coding genes examined was subject to neutral evolution [31].

Based on the above data, only possible differences in photosynthetic efficiency could be expected within the divergence between the individual variants considered in this section, but such assumptions should be confirmed by further appropriately designed biochemical studies.

### 5.2. Phylogenetic Relationships

Overall, the results [31] demonstrated a high degree of conservation of the analyzed material, with the level of variation among the nine *D. gratianopolitanus* cultivars proving comparable to interspecific variation. Analyses examining the phylogenetic relationships within 16 *Dianthus* species [32] allowed for the determination of the affiliation to two sister clades, A and B. Clade A included *D. gratianopolitanus*, *D. caryophyllus*, *D. barbatus*, and two cultivated varieties (“HY” and “WC”), while clade B contained *D. chinensis*, *D. superbus*, *D. longicalyx*, and the F1 “87M”. The latter was the hybrid offspring of the F1 “HY” and *D. chinensis*. It should be emphasized that analyses of hypervariable regions and sequence divergence allowed for the selection of a limited number of regions with more divergent sequences, which ultimately led to the achievement of characteristic DNA markers, at least one of which could be the basis for assigning individual accessions to clades. This contributed not only to the classification of *Dianthus* taxa, but also shed light on the evolutionary pathways of cpDNA [32].

Taken together, these results suggest that *D. gratianopolitanus* and *D. caryophyllus* were most closely related. It is therefore clear that hybridization between individual *Dianthus* species results in the indistinct genetic background, and the demonstrated differences, albeit slight, may serve as a basis for further genetic research on the delimitation within the genus [31,32].

In another study [57], Fassou et al. (2022) stated that within clade A—which encompasses a large proportion of the Euro-Mediterranean representatives of the genus *Dianthus*—there were well-supported subclades associated with specific taxa and geographically proximate populations. At the same time, samples of *D. gratianopolitanus* were scattered throughout this clade. The authors emphasized that a deep division involving various plastid haplotypes was observed in this species. This means that the phylogenetic analysis did not yield a pattern of “population A → *D. gratianopolitanus* → a single, cohesive branch,” but rather “various populations of *D. gratianopolitanus* → distinct lineages within the larger clade A”. Notably, no distinct morphological differences were found among the analyzed individuals or populations. The study utilized two types of molecular data. The first was cpDNA. A combined dataset of plastid regions, *matK–trnK–psbA*, *rpl32–trnL*, and *trnQ–rps16*, was analyzed. In total, the plastid matrix comprised 202 taxa and 5573 positions, including 5297 nucleotide characters and 287 coded indels (insertion/deletion, i.e., the insertion or deletion of a DNA fragment). It is precisely the plastid data that reveals a particularly interesting picture of *D. gratianopolitanus*: individuals from different populations appear in distinct sub-lineages. The second type was nuclear DNA, specifically, the ITS (Internal Transcribed Spacer), in this case, nrITS region (nuclear ribosomal ITS). The ITS matrix included 136 taxa and spanned 714 nucleotides. However, the authors noted that the ITS tree lacked resolution, exhibiting a large polytomy within the main *Dianthus* clade. ITS provided significantly lower resolution than the plastid data. Consequently, the most crucial information regarding *D. gratianopolitanus* stems from the phylogeny based on plastid DNA [57].

The authors of [57] generally concluded that genetic distances within the genus *Dianthus* are very small, which limits the ability to resolve interspecific relationships at a phylogenetic level. They interpreted this as a possible result of rapid evolutionary radiation—the rate of taxon differentiation may have outpaced the accumulation of mutations in the analyzed DNA regions [57].

This phylogenetic pattern is particularly notable in the case of *D. gratianopolitanus*. Geographically distinct populations did not form a single, cohesive lineage, and their plastid haplotypes were distributed across various parts of the clade; yet, the populations remained morphologically similar. This implies that molecular variation does not straightforwardly correspond to morphological differentiation, and that species boundaries and evolutionary history are not fully resolved by the plastid markers analyzed in this case [57].

## 6. Interdisciplinary Research, Knowledge Gaps and Future Directions

Of course, the issues discussed here do not exhaust all the scientific questions that arise when studying carnation biology. However, these are key questions arising from the knowledge accumulated so far. As is often the case in science, the more knowledge there is, the more questions and uncertainties there are. The focus here is on presenting the perspective of interdisciplinary research, bringing together botanists working with diverse methods.

### 6.1. From Habitat Description to Mechanism

*Dianthus gratianopolitanus* has been extensively described in terms of habitat preferences, yet the ecological picture that emerges is paradoxical. When each environmental factor is examined separately—soil chemistry, light availability, temperature, and precipitation—the species appears to tolerate a relatively wide range of conditions. At the same time, *D. gratianopolitanus* is a rare species that, according to the literature, occurs in highly specific microhabitats. It points out that in some regions, it is found on south-facing slopes in shade and on north-facing slopes in full light [2]. This highlights the importance of temperature as an integrating variable, itself shaped by the height above sea level, slope aspect, canopy cover, and amount and seasonal distribution of rainfall. These observations suggest that we may be dealing with phenomena well known in plant ecology: resource compensation (the plant offsetting the influence of one factor by another) or microhabitat compensation (different combinations of conditions producing a similar net ecological effect) [58,59]. Phenotypic plasticity—the capacity of an organism to adjust its functioning in response to environmental variation—and its limits may play a central role in species survival [60]. The experimental evidence suggests that phenological and reproductive responses of *D. gratianopolitanus* to increasing temperature sums are driven primarily by plastic responses rather than by genetic differentiation among populations [7]. The species’ ability to persist under varied cultivation conditions ex situ further suggests that its rarity in the wild reflects competitive interactions, dispersal constraints, or specific microhabitat requirements rather than strict physiological limits. To understand what conditions *D. gratianopolitanus* truly requires, greater insight into its physiological mechanisms and its biochemical responses to environmental stress is needed.

Very interesting research has been carried out looking for adaptations of specimens living on different types of bedrock by Schöne (2025) [8]. Amplified Fragment Length Polymorphism, so-called AFLP methodology, was used to compare the genetic research differentiation between plants originated from calcareous bedrock and siliceous bedrock (whereby volcanic and sandy bedrock were combined in a siliceous group). It highlights the possible influence of edaphic factors on the differentiation and adaptation of *D. gratianopolitanus* [8]. The author also showed that plants from calcareous bedrock invested more in vegetative growth, while plants with siliceous origin were characterized by higher flowering. It can be associated with the fact that the first ones have adapted to higher competitiveness for space on the spatially restricted limestone outcrops (vegetative reproduction can be advantageous in that situation), and the second ones adapted to faster colonization of the more open habitats by seed production. Schöne (2025) also conducted experiments showing that *D. gratianopolitanus* from calcareous and siliceous bedrock differ in element accumulation [8]. Therefore, the question arises about the physiology differences in plants originating from sites with calcareous and siliceous bedrock types. Additionally, it should be noted that the substrates in experiments conducted by Schöne (2025 [8]) have a neutral to slightly alkaline pH value. Seed germination studies also revealed that the species prefers this pH. Meanwhile, in Poland, this species grows in pine forests, habitats characterized by acidic soil pH. As pH influences the ability to absorb mineral nutrients and levels of possibly toxic elements, it would be worthwhile to compare the physiology of plants growing in these acidic habitats compared to alkaline habitats.

Whether the observed differences between plants originated from different bedrocks that represent genetically fixed local adaptations (true ecotypes) or phenotypic plasticity expressed in response to substrate chemistry has not been fully resolved. This question is more than academic. If calcareous and siliceous populations represent locally adapted ecotypes, then translocation of plants between substrate types—for example, using material from German limestone populations to augment Polish sandy-soil populations—risks introducing maladapted genotypes. If the differences are purely plastic, such translocations would be genetically safe. Reciprocal transplant experiments, combined with genome-wide SNP scans (e.g., using RADseq or ddRAD approaches) and environmental association analyses, would distinguish these alternatives. FST-outlier analysis could identify loci under divergent selection between substrate types, while environmental association analysis (EAA) could link allele frequencies at specific loci to soil pH, calcium availability, and other edaphic variables.

Kolb (2000) demonstrated a significant impact of climate change on nutrient balance in the soils of forest ecosystems, which is particularly relevant here because the studied species occurs in the lowland pine forests [61]. Earlier, this issue was examined in other ecosystem types, including mountain grasslands [62]. Additional nitrogen is at least partially absorbed by vegetation, potentially altering competitive relationships and thereby affecting the carnation, which appears to be a relatively weak competitor. Recent studies indicate that soil nitrogen enrichment may be an important factor shaping plant distribution ranges in Europe [63]. According to the meta-analysis by He et al. (2025), nitrogen addition can significantly influence phenological stages [64]. Nitrogen deposition may also operate through physiological mechanisms that increase plant susceptibility to fungal pathogens [65]. However, none of these analyses specifically considered *D. gratianopolitanus*. These additional factors, which complicate the projections proposed by Schöne (2025) regarding the impact of climate change on the carnation’s range, warrant further investigation [8].

### 6.2. From Genes to Function

The chloroplast genomic analyses reviewed in Section 4 identified four genes under positive selection across all nine *D. gratianopolitanus* accessions: *atpB*, *ndhF*, *psbB*, and *ycf1* [31]. These are not random genes. The *atpB* gene encodes the β subunit of the chloroplast ATP synthase—the enzyme that converts the proton gradient generated by light reactions into ATP, the energy currency of the cell. The *ndhF* gene encodes a subunit of the NADH dehydrogenase complex, which mediates cyclic electron flow around PS I and is particularly important for stress protection under drought, high light, and temperature extremes [47,48]. The *psbB* gene encodes the CP47 chlorophyll-binding protein of Photosystem II, a core component of the light-harvesting and charge-separation machinery. The *ycf1* gene encodes a component of the chloroplast protein import apparatus [51].

The functional logic of positive selection in precisely these genes is compelling: a species inhabiting exposed rock outcrops, subject to intense solar radiation, episodic drought, and extreme temperature fluctuations, would benefit from adaptive variation in the very machinery responsible for light harvesting, electron transport, ATP synthesis, and stress-responsive cyclic flow. However, the chloroplast genomic data alone cannot confirm this interpretation. The Ka/Ks ratio identifies selection at the sequence level; it does not demonstrate that sequence variation translates into functional differences in protein performance, or that these functional differences are ecologically relevant.

The critical missing link is physiological measurement. Do accessions with different *psbB* or *atpB* variants differ in their photosynthetic efficiency (Fv/Fm, electron transport rate, maximum carboxylation rate), their response to high light stress, or their drought tolerance? Gas exchange measurements (A_max_, stomatal conductance, water-use efficiency) and chlorophyll fluorescence analysis across accessions from climatically contrasting populations would begin to answer this question. Such measurements are entirely feasible with standard equipment (LI-COR gas exchange systems, pulse-amplitude modulation fluorometers) and would represent the first direct bridge between the chloroplast genomic data and the ecological reality of the species’ habitat.

The observed genetic variation, particularly in photosynthesis-related genes under positive selection, suggests that *D. gratianopolitanus* accessions may differ in photosynthetic efficiency, stress tolerance, or resource-use strategies. These physiological differences could underlie variation in habitat preferences or performance across the species geographic range—for example, enabling persistence on nutrient-poor, drought-prone substrates. However, these hypotheses require validation through comparative physiological experiments measuring photosynthetic rates, water-use efficiency, and stress responses across genetically characterized accessions.

### 6.3. From Population Biology to Conservation Perspectives

Despite decades of field observation, no multi-year demographic study providing age- or stage-structured life tables, population growth rates (λ), or elasticity analyses has been published for *D. gratianopolitanus*. Consequently, it remains unknown whether any given population is stable, growing, or declining, or which life stage—seedling establishment, adult survival, or seed production—most constrains population growth. The descriptive evidence strongly suggests that seedling recruitment is the most precarious phase: Jackel (1999, cited in [2]) documented high seedling mortality attributable to summer drought, and field monitoring at Swabian Jura sites found no significant seedling establishment over a five-year period (2015–2020), suggesting that natural recruitment may currently be insufficient to compensate for adult mortality [7]. Whether seedling recruitment or adult survival contributes more to population persistence—a question with direct management implications—can only be answered through formal demographic modeling [66].

Applying standard demographic methods to *D. gratianopolitanus*, however, presents particular challenges inherent to the species’ biology. The approach requires age- or stage-structured life tables describing the survival, growth, and reproduction of individuals through time. This is exceptionally difficult for a species that reproduces vegetatively through lateral vegetative spread, making it difficult to delimit individual genets or assign developmental stages in the field. Beyond the seedling stage, the only other potentially distinguishable stage is the transition between vegetative and reproductive rosettes, though the reversibility of this transition complicates its use as a demographic category. Under natural population conditions, determining the boundaries between individuals—let alone their ages—is therefore not feasible with standard field methods. A realistic path forward would combine controlled laboratory experiments—tracking individual ramet production rates, clonal spread, and longevity under defined conditions—with field-based methods for estimating population growth rates without individual tracking.

Both processes—long-term persistence through vegetative expansion and episodic seedling recruitment when suitable microhabitats become available—undoubtedly contribute to population maintenance. What remains unknown is the relative effectiveness of each, and crucially, how this balance may shift under changing climatic conditions. Given that increasing temperature sums already demonstrably reduce the seed set and compress the flowering window [7], and that summer drought is a documented cause of seedling mortality (Jackel, 1999, cited in [2]), the sexual recruitment pathway appears particularly vulnerable to climate warming. Quantifying the demographic contribution of each process would therefore provide not merely academic insight, but practical guidance for conservation management—informing decisions about whether to prioritize habitat interventions that open new establishment microsites, or population support strategies that compensate for declining reproductive output.

The species sheds seeds in mid-to-late summer, following a flowering period from late May to late June (Seybold, 1990, cited in [2]). Laboratory experiments demonstrate that germination is non-dormant—seeds germinate immediately when conditions are suitable—and that germination success is strongly temperature dependent [19]. This raises a question that has not been resolved in the field: do seeds shed in summer germinate in the same season, or do they overwinter and germinate the following spring? The laboratory evidence is ambiguous. Warm summer temperatures are favorable for germination, and seeds are non-dormant. Summer germination is therefore physiologically possible. However, seedlings establishing in late summer would face immediate drought stress and potentially winter desiccation before developing an adequate root system. Spring germination, by contrast, would allow seedlings to develop during a period of more moderate temperatures and, in many parts of the range, higher soil moisture. This question has implications for analyzing the impact of climate change on a species’ ability to survive: if recruitment is strictly limited to spring, warmer, drier springs will potentially increase the risk of extinction for small populations. We also do not know whether *D. gratianopolitanus* maintains a persistent soil seed bank, which would enable recruitment due to the episodic drought years.

Direct conservation relevance also has haplotype geography. Populations carrying unique haplotypes not found elsewhere represent irreplaceable components of the species’ evolutionary heritage. If the UK population, for example, carries haplotypes absent from continental populations, it constitutes a distinct evolutionary unit deserving independent conservation status. Conversely, if populations share haplotypes across national boundaries, coordinated cross-border management may be more effective than country-by-country approaches. Overlaying haplotype distributions with species distribution model (SDM) projections of climatically stable areas would identify populations that are both genetically distinct and likely to persist under projected climate change—a doubly-prioritized target for conservation.

The AFLP data of Schöne (2025) and Putz et al. (2015) indicate that isolated populations maintain genetic variation comparable to larger, more connected ones—a finding consistent with the species’ apparent adaptation to functioning under fragmentation [8,18]. However, genetic diversity measured by neutral markers (AFLPs) does not necessarily reflect adaptive diversity. Small, isolated populations may retain neutral diversity through historical legacy while losing adaptive variation through drift—particularly variation at loci under positive selection, such as those identified in the chloroplast genome.

A key question for conservation prioritization is whether the genetically most distinct populations (those with the highest AFLP divergence or unique haplotypes) are also the demographically most vulnerable (smallest in size, most isolated, subject to the greatest habitat degradation). If genetic uniqueness and demographic vulnerability coincide, conservation resources are most efficiently directed at these populations. If they diverge—that is, if the most genetically distinct populations are also the most robust demographically—then prioritization based on genetic criteria alone may be misleading. Combining demographic monitoring data with genetic characterization across a representative sample of populations would resolve this question and provide a rational basis for conservation prioritization.

Genomic offset—the predicted mismatch between a population’s current genetic composition and the genetic composition that would be optimal under future climatic conditions—represents a potentially powerful tool for identifying populations at risk of maladaptation before demographic decline becomes detectable [67]. Calculating genomic offset requires genome-wide SNP data (currently unavailable for *D. gratianopolitanus*) and environmental association analyses linking allele frequencies at specific loci to climatic variables. Once such data become available, genomic offset modeling could identify populations whose current genetic composition is already poorly matched to projected future climates—providing an early warning system that operates on a faster timescale than demographic monitoring. The combination of genomic offset with SDM projections would be particularly powerful: populations predicted to experience both high climatic unsuitability (SDM) and high genomic offset (genetic mismatch) would face a double jeopardy of habitat loss and genetic maladaptation, and would represent the most urgent conservation priorities.

It is important to mention here that difficulties in population studies affect also possibilities of estimation of minimum viable population size (MVP)—the population size below which the extinction risk becomes unacceptably high. Many of the known populations of *D. gratianopolitanus* are small, and whether they exceed their MVP is unknown. This is another case which makes long-term population monitoring and further genetic analysis essential.

### 6.4. From In Vitro Propagation to Genotype-Informed Reintroduction

The conservation strategies outlined in Section 6.3—including population reinforcement, assisted gene flow, and the establishment of ex situ reserves—ultimately depend on the availability of clonally faithful, genetically characterized plant material in sufficient quantities. *Dianthus gratianopolitanus* occupies a narrowing mosaic of calcareous rock outcrops and semi-open grasslands that are increasingly isolated from one another, making natural recruitment insufficient to compensate for ongoing population losses across much of its range. The pressing practical question is therefore not only which populations should be the focus of conservation action, but also how viable plant material can be reliably produced and transferred to target sites. Encouragingly, micropropagation protocols for *D. gratianopolitanus* have been established and demonstrated to yield both high rooting rates and long-term genetic stability, indicating that the technical prerequisites for ex situ propagation are largely in place. The central unresolved challenge lies a step further: translating these propagation capabilities into genotype-informed reintroduction programs, in which source populations are selected on the basis of the genetic differentiation patterns discussed in Section 6.3, and material is allocated to recipient sites with appropriate regard for local adaptation and the avoidance of outbreeding depression. The following subsection summarizes the in vitro protocols currently available for *D. gratianopolitanus* that would underpin any such program.

Fraga et al. [68] aimed to develop an efficient in vitro propagation protocol for *D. gratianopolitanus* cultivars ‘Spotti’ and ‘Frosty Fire’. The authors tested various explant types—shoot tips, nodes, leaves, and ovaries—obtained from virus-free stock plants maintained under greenhouse conditions, along with different growth regulator combinations, to determine optimal conditions for shoot induction, rooting, and acclimatization. Cultures were established on Murashige and Skoog (MS) basal medium. The cytokinins tested included 6-benzylaminopurine (BAP), kinetin, and 2-isopentenyladenine (2-iP); the auxins tested included indole-3-acetic acid (IAA), 1-naphthaleneacetic acid (NAA), and indole-3-butyric acid (IBA). The research team used hormone concentrations in various combinations, typically in the micromolar range (e.g., 0.57 µM IAA, 2.46 µM 2-iP). The key results are summarized in Table 5.

Fraga et al. [68] and Sarropoulou et al. [69] demonstrated that *D. gratianopolitanus* can be micropropagated effectively using simple media and minimal growth regulators. Ex vitro rooting is superior to in vitro rooting, providing 97% success without exogenous auxins. Acclimatization under controlled humidity and temperature conditions resulted in over 99% survival. What should be emphasized is that the protocol offers a reliable method for commercial propagation and conservation of *D. gratianopolitanus*.

The micropropagation of *D. gratianopolitanus* Vill., a rare Alpine species, has been successfully achieved as part of conservation-oriented in vitro studies on endangered *Dianthus* taxa. The research conducted by Cristea et al. [70] aimed to establish efficient conservative micropropagation protocols to ensure the long-term ex situ preservation of endemic *Dianthus* species. The experiments used explants derived from healthy donor plants cultivated under controlled conditions. Shoot tips and nodal segments were the primary sources of explants, as they showed the highest regeneration potential. Cultures were initiated on MS basal medium supplemented with BAP as the cytokinin and NAA as the auxin. The combination of 0.5 mg L^−1^ BAP and 0.1 mg L^−1^ NAA proved optimal for the induction of multiple shoots [70].

In shoots, proliferation was enhanced by subculturing on the same medium every 4–5 weeks. The multiplication rate remained high and stable over several passages without significant morphological abnormalities, indicating genetic stability and the high regenerative capacity of the material. For the rooting stage, regenerated shoots were transferred to MS medium containing IBA at concentrations between 0.5 and 1.0 mg L^−1^. Rooting percentages exceeded 90%, producing well-developed root systems suitable for acclimatization [70].

Rooted plantlets were successfully acclimatized under greenhouse conditions, with a survival rate over 95% after transfer to soil substrate composed of peat and perlite (1:1). The regenerated plants maintained normal morphology and were phenotypically identical to the donor material, confirming the reliability of the in vitro propagation system [70]. The protocol developed for *D. gratianopolitanus* can contribute to the conservation of its genetic diversity and to the reduction of extinction risk [70,71].

## 7. Conclusions

**Figure 2:**
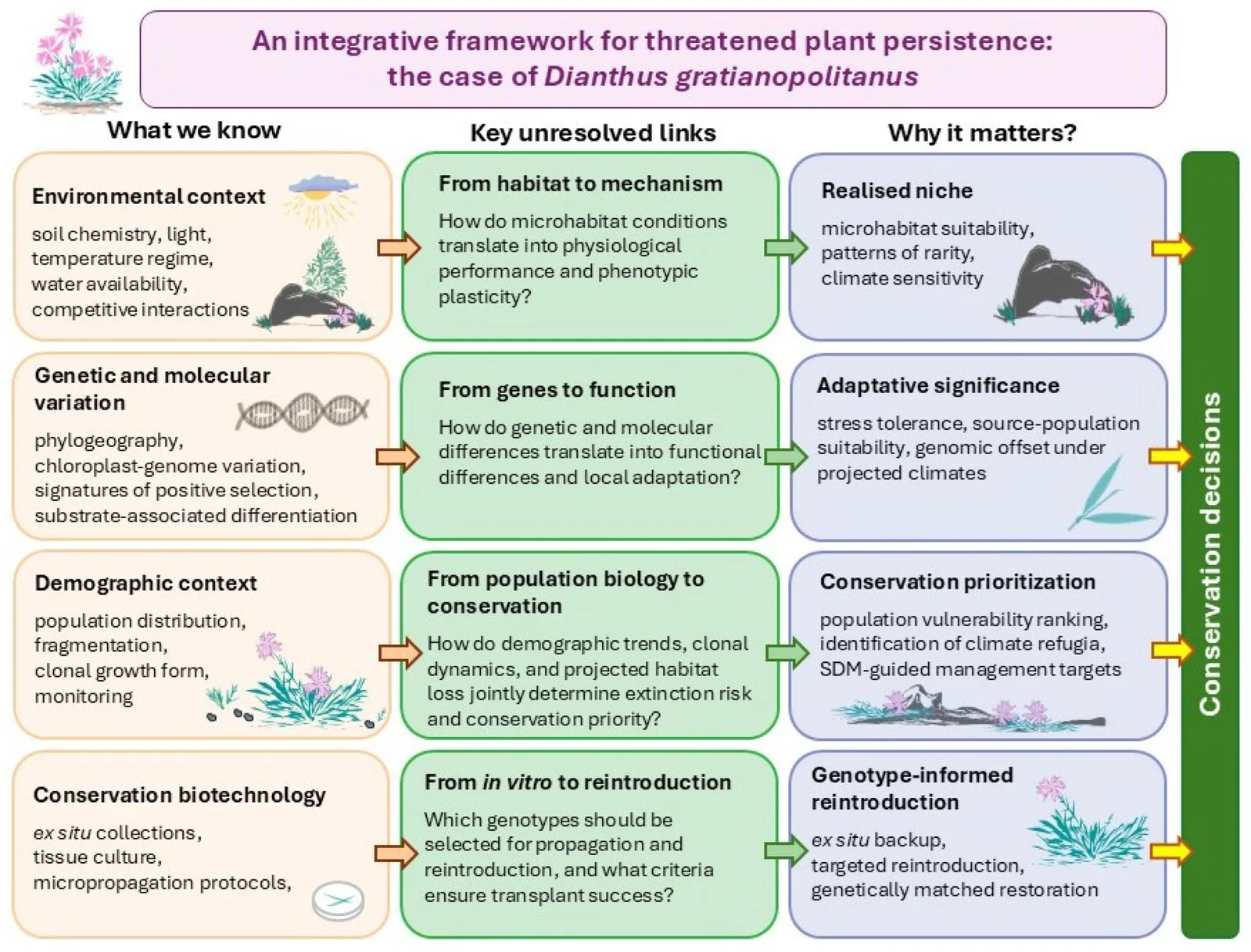
An integrative framework for research-driven conservation of *Dianthus gratianopolitanus*. The figure organizes current knowledge and future research priorities across four thematic levels, each corresponding to a major section of this review: (1) environmental context and ecophysiology, (2) genetic and molecular variation, (3) demographic context and population biology, and (4) conservation biotechnology. For each level, the left column summarizes established knowledge, the central column identifies the key unresolved mechanistic link that limits conservation-relevant understanding, and the right column specifies why resolving that link matters for species persistence. The vertical banner on the right indicates that advances at all four levels collectively inform conservation decisions for this species.

Effective conservation of *Dianthus gratianopolitanus* requires the coordinated integration of ecological, physiological, genetic, demographic, and biotechnological research approaches (Figure 2).

The analysis of currently available knowledge reveals not only what has been established, but also where the most consequential gaps lie and which methodological strategies are best positioned to address them.

- *D. gratianopolitanus* combines apparent ecological flexibility with strong microhabitat dependence, suggesting that phenotypic plasticity, microhabitat compensation, and competitive dynamics jointly shape local abundance; the key unresolved question is how specific combinations of soil chemistry, light regime, water availability, and competitive pressure translate into physiological performance and, ultimately, into the species’ realized niche. Ecophysiological experiments along natural microhabitat gradients would provide the mechanistic foundation needed to predict population responses to ongoing climate change.
- Genetic and phenotypic differentiation among populations on contrasting substrates points to a role of edaphic adaptation in divergence, but the relative contributions of genetic fixation, phenotypic plasticity, and phylogeographic history remain poorly resolved. Integrating reciprocal transplant experiments with landscape genomic analyses would identify which populations harbor locally adaptive variation and which already face genetic maladaptation under projected climates, providing essential information for conservation prioritization.
- Chloroplast genes under positive selection are enriched in photosynthesis and stress-response functions, suggesting molecular adaptation to drought, high irradiance, and temperature extremes. However, the ecological significance of these signatures remains to be validated: it is not yet established whether the identified variants actually confer measurable physiological advantages under field conditions. Comparative physiology linking the chloroplast genotype to performance under controlled stress, complemented by transcriptomics or proteomics, represents the most direct route toward this validation and could also yield molecular markers for selecting physiologically suitable source material in conservation translocations.
- The absence of long-term demographic data is one of the most critical knowledge gaps for *D. gratianopolitanus*, and standard matrix-model approaches are difficult to apply because the clonal growth form renders the delimitation of individuals and the identification of distinct life stages problematic in field settings. Monitoring frameworks that integrate physiological condition indices, molecular estimates of effective population size, and demographic models adapted for clonal plants may provide a more realistic basis for population viability assessment.
- Combining genome-wide marker data with species distribution modeling (SDM) offers the clearest pathway from research to conservation management: SDM projections identify populations facing the greatest habitat loss under future climates, while genomic offset analysis reveals those already genetically mismatched to projected conditions, with doubly vulnerable populations representing the highest conservation priority.
- Established in vitro micropropagation protocols for *D. gratianopolitanus* and related *Dianthus* species provide a practical foundation for ex situ conservation and planned reintroduction, enabling the maintenance of viable material from genetically characterized individuals. Establishing explicit criteria for genotype-informed reintroduction would transform in vitro propagation from a precautionary backup into a strategically targeted conservation tool.

The six directions outlined above are deeply interconnected: mechanistic understanding of ecophysiology provides the context for interpreting genomic patterns; demographic models are more reliable when parameterized with genetic data; and decisions about reintroduction depend on knowledge spanning all preceding levels. The conceptual and methodological framework developed here may serve as a template for similarly structured reviews of other threatened plant taxa where conservation decisions must be made under deep uncertainty.

## Figures and Tables

**Table 1:** Inclusion and exclusion criteria used for the systematic literature search on *D. gratianopolitanus*.

Criteria	Inclusion	Exclusion
Taxonomic scope	Studies concerning *Dianthus gratianopolitanus* Vill. or its taxonomic synonym *Dianthus caesius*	Studies concerning other *Dianthus* species without species-specific information on *D. gratianopolitanus*
Research topic	Taxonomy, phylogeny, distribution, biogeography, ecology, habitat, population biology, genetics, reproduction, pollination, germination, physiology, adaptation, climate change, conservation, reintroduction, restoration, molecular biology and genomics	Studies unrelated to the biology, ecology, genetics, distribution or conservation of the species
Species-specific information	Studies providing information that could be specifically attributed to *D. gratianopolitanus*	Studies in which information concerning *D. gratianopolitanus* could not be separated from other taxa
Publication type	Peer-reviewed research articles, reviews, dissertations, monographs, red lists, conservation guidelines, floristic and ecological reports, and other authoritative sources containing relevant species-specific information	Unverified sources, duplicate publications, conference abstracts without sufficient data, and sources lacking relevant scientific or conservation information
Availability of information	Publications providing sufficient bibliographic and/or full-text information to evaluate their relevance and findings	Publications for which sufficient information was unavailable
Date	Publications available up to and including 7 October 2026	Publications published or indexed after 7 October 2026
Molecular databases	NCBI nucleotide records and sequence information relevant to *D. gratianopolitanus*; BLAST results used for sequence identification and comparison	Sequence records unrelated to *D. gratianopolitanus* or lacking sufficient information for reliable identification

**Table 2:** Summary of chloroplast genome data for *D. gratianopolitanus* varieties. cpDNA size and GC content varied slightly among accessions, indicating a high level of conservation [31].

Variety	NCBI Accession No.	cpDNA Size (bp)	GC Content (%)	No. of Genes	Protein-Coding Genes	No. of SSRs
*D. gratianopolitanus* dg1602	LN877389	149,474	36.29	124	84	142
*D. gratianopolitanus* dg1869	LN877391	149,735	36.33	125	84	150
*D. gratianopolitanus* dg3051	LN877395	149,612	36.31	124	84	141
*D. gratianopolitanus* dg2134	LN877392	149,708	36.32	125	84	149
*D. gratianopolitanus* dg1856	LN877390	149,499	36.30	124	84	144
*D. gratianopolitanus* dg2769	LN877393	149,691	36.32	124	84	142
*D. gratianopolitanus* dg1255	LN877387	149,563	36.31	124	84	140
*D. gratianopolitanus* dg1370	LN877388	149,614	36.30	124	84	146
*D. gratianopolitanus* dg2868	LN877394	149,524	36.30	124	84	146

**Table 3:** Repeat structure and SSR characteristics of *D. gratianopolitanus*; SSR patterns were similar across accessions, and only minor differences were observed compared with other species [31].

SSR Type	Frequency Range	Most Common Motifs	Notes
Mononucleotide	55–67	A/T repeats	Most abundant
Dinucleotide	55–67	AT, TA	Highly frequent
Trinucleotide	≈4	Variable	Rare
Tetranucleotide	≈4	Variable	Rare
Pentanucleotide	Low	–	Least represented
Hexanucleotide	1 copy (dg1602, dg2769, dg3051)	–	Absent in *D. caryophyllus*

**Table 4:** Genes showing sequence variation in *D. gratianopolitanus* [31].

Gene	Function	Location/Variant	Observation	References
*psbA*	Photosystem II D1 protein	LSC, dg1869	1062 bp difference; electron transport	[31,33]
*trnH*	tRNA for histidine	Other cultivars	Variant tRNA site	[31,34]
*atpF*	ATP synthase subunit I	dg1255, dg1370	Major variation	[31,35]
*clpP*	ATP-dependent Clp protease subunit	dg1602, dg2868	Strong variation	[31,36]
*petD*	Cytochrome b_6_f subunit IV	dg3051	Minor differences	[31,37]
*ycf3*	PSI assembly factor	dg3051	High divergence	[31,38]
*rpl16*	50S ribosomal protein L16	all tested *D. gratianopolitanus* varieties	Stable (vs. aberrant in *D. caryophyllus*)	[31,39,40]
*rps19*	30S ribosomal protein S19	all tested *D. gratianopolitanus* varieties	Conserved, LSC/IR border	[31,41]
*ycf1*	Hypothetical protein, IRb region	all tested *D. gratianopolitanus* varieties	Same length across varieties; +3 bp in *D. caryophyllus*	[31]

**Table 5:** Results of micropropagation and acclimatization of *D. gratianopolitanus* [68].

Stage	Medium/Treatment	Cultivar	Result	Observation
Shoot induction	MS + 0.57 µM IAA	‘Spotti’	Highest number of shoots from shoot tips	Best response from apical meristems
	MS + 2.46 µM 2-iP	‘Spotti’	Good shoot regeneration from nodes	Higher cytokinin levels caused some hyperhydricity
	MS without PGRs	‘Frosty Fire’	Highest multiplication rate	No hormones needed for optimal growth
Explant type comparison	Shoot tips and nodes	Both cultivars	80–90% regeneration	Efficient sources for propagation
	Leaves and ovaries	Both cultivars	<10% regeneration	Poor morphogenetic response
Rooting (in vitro)	MS with IBA, NAA, or IAA (0.5–1 mg L^−1^)	Both cultivars	No significant differences in root number among auxins	Confirms neutral effect of auxin type
Rooting (ex vitro)	Hormone-free substrate	Both cultivars	97% rooting success	Highest efficiency obtained ex vitro
Acclimatization	Commercial substrate, 80% RH, 20 °C ± 4 °C (day)/16 °C ± 4 °C (night)	Both cultivars	>99% survival in greenhouse	Very high acclimatization rate

## Data Availability

No new data were created or analyzed in this study. Data sharing is not applicable to this article.

## Abbreviations

The following abbreviations are used in this manuscript:

2-iP	2-isopentenyladenine
AFLP	Amplified Fragment Length Polymorphism
atpB	ATP synthase β subunit
atpF	ATP synthase subunit I
BAP	6-benzylaminopurine
BIO1–BIO19	Bioclimatic variables
clpP	ATP-dependent Clp protease subunit
cpDNA	Chloroplast DNA
EAA	Environmental Association Analysis
ht16, ht19	haplotype
IAA	indole-3-acetic acid
IBA	indole-3-butyric acid
IRs	Inverted Repeat regions
ITS	Internal Transcribed Spacer region
nrITS	nuclear ribosomal ITS
LSC	Large Single-Copy region
MVP	Minimum Viable Population size
NAA	1-naphthaleneacetic acid
ndhF	NADH dehydrogenase subunit F
petD	Cytochrome b_6_f subunit IV
PS I	Photosystem I
PS II	Photosystem II
psbA	Photosystem II D1 protein
psbB	CP47 chlorophyll-binding protein
RSCU	Relative Synonymous Codon Usage
RADseq	Restriction-site Associated DNA Sequencing
rpl16	50S ribosomal protein L16
rps19	30S ribosomal protein S19
SSC	Small Single-Copy region
SSRs	Simple Sequence Repeats
SDM	Species Distribution Modeling
TIC	Translocon at the Inner Chloroplast membrane
trnH	tRNA for histidine
ycf1	Hypothetical protein, IRb region
ycf3	PSI assembly factor
